# Gamified mHealth System for Evaluating Upper Limb Motor Performance in Children: Cross-Sectional Feasibility Study

**DOI:** 10.2196/57802

**Published:** 2025-02-28

**Authors:** Md Raihan Mia, Sheikh Iqbal Ahamed, Samuel Nemanich

**Affiliations:** 1 Department of Computer Science Marquette University Milwaukee, WI United States; 2 Department of Occupational Therapy Marquette University Milwaukee, WI United States

**Keywords:** mobile health, mHealth, digital health, mobile apps, smartphones, iPad, gamification, serious games, digital interventions, digital technology, spatiotemporal, upper limb movement, motor performance, motor skills, pediatrics, toddler, children, youth

## Abstract

**Background:**

Approximately 17% of children in the United States have been diagnosed with a developmental or neurological disorder that affects upper limb (UL) movements needed for completing activities of daily living. Gold-standard laboratory assessments of the UL are objective and precise but may not be portable, while clinical assessments can be time-intensive. We developed MoEvGame, a mobile health (mHealth) gamification software system for the iPad, as a potential advanced technology to assess UL motor functions.

**Objective:**

This feasibility study examines whether MoEvGame can assess children’s whole-limb movement, fine motor skills, manual dexterity, and bimanual coordination. The specific aims were to (1) design and develop novel mHealth gamified software tools to examine theory-driven features of UL movement, (2) analyze spatiotemporal game data with new algorithms and statistical techniques to quantify movement performance as a parameter of speed, accuracy, and precision, and (3) validate assessment methods with healthy participants from schools.

**Methods:**

Elementary school children (N=31, median 9.0, IQR 4.0-14.0 years old) participated by playing 5 games. The game tasks were focused on key features of skilled motor control: (1) whole limb reaching, (2) fine motor control and manual dexterity, and (3) bilateral coordination. Spatiotemporal game data were transferred and stored in a cloud-based data management server for further processing and analysis. We applied change point detection (ie, the pruned exact linear time method), signal processing techniques, and other algorithms to calculate movement speed and accuracy from spatiotemporal parameters. Different statistical methods (ie, Pearson correlation, mean, standard deviation, *P* value, 95% confidence interval) were used to compare speed-accuracy tradeoffs and evaluate the relationship between age and motor performance.

**Results:**

A negative correlation was identified between speed and accuracy in the whole limb movement (*r*=–0.30 to –0.42). Significant relationships between age and upper limb performance were found: older participants exhibited lower errors with faster completion times compared to younger participants. Significant differences in bimanual coordination were found related to phase synchronization (in-phase congruent [mean 28.85, SD 18.97] vs antiphase congruent [mean 112.64, SD 25.82] and in-phase mirrored [mean 23.78, SD 16.07] vs antiphase mirrored [mean 121.39, SD 28.19]). Moreover, the average speed (revolutions per second) and travel distance (m) of the in-phase mode were significantly higher than those of the antiphase coordination.

**Conclusions:**

Results of this feasibility study show that spatiotemporal data captured from the mHealth app can quantify motor performance. Moving beyond traditional assessments, MoEvGame incorporates gamification into ubiquitous and accessible technology as a fast, flexible, and objective tool for UL motor assessment.

## Introduction

### Background

Upper limb (UL) movements such as reaching, grasping, and finger individuation are fundamental to a child’s development and to becoming independent with activities of daily living. Furthermore, coordinated and purposeful UL movement enables participation in play and leisure activities. In the United States, more than 17% of children (1 in 6) aged 3-17 years were diagnosed with a developmental disability [1], many of which affect UL motor function and limit independence and quality of life. Importantly, motor impairments, if left unaddressed, will lead to chronic motor disabilities in adulthood that are linked to poor health outcomes [2-4].

To aid rehabilitation professionals such as physical and occupational therapists, timely and objective assessments of a child’s motor abilities are pivotal to establishing treatment plans and monitoring the impact of ongoing neurorehabilitation interventions. Standardized play-based tests of gross and fine motor function are used clinically to assess motor outcomes [5-7]; however, these tests may have subjective scoring and require time to administer, some up to one hour, which challenges a child’s motivation and attention. More objective laboratory tests use 3D motion capture technology for kinematic analysis of the UL movement. However, this laboratory equipment is expensive, and while newer portable solutions have been developed, it is usually restricted to a laboratory environment [8]. Furthermore, both laboratory and clinical assessments require in-person sessions wherein clinicians or researchers directly evaluate participants; such in-person visits can be a major limitation for families with travel, transportation, or time restrictions and thus may decrease research participation altogether [9]. Therefore, the current landscape of UL motor assessments shows gaps in meeting the needs of clinicians and researchers to be objective and the needs of families or participants to be flexible and accommodating [10,11].

### Related Work

Mobile health (mHealth) platforms have the potential to address these gaps by integrating mobile technology with gamified software applications to test UL motor functions [12-15]. First, mHealth technologies are portable and would enable data collection in any setting with wireless or network connectivity. Second, they are accessible, with recent estimates indicating that a large majority of Americans, even those from underrepresented racial, ethnic, and socioeconomic groups, own and use mobile technology [16]. Lastly, they are customizable, primarily via flexibly programmed software packages that allow for many experimental tasks to be implemented seamlessly in a mobile app [17]. A recent survey on the feasibility and acceptability of mHealth technologies in clinical trials of movement disorders (n=54, by searching ClinicalTrials.gov) revealed the potential of these technologies to increase participation, reduce the cost and duration of procedures, overcome barriers to collect and validate data, and enable real-time monitoring of outcomes [18].

Specific to studying UL movement and motor control, two examples in the literature provide a framework for expanding experiments outside of the laboratory using mHealth approaches. First, the portable motor learning laboratory was developed to simulate lab equipment needed to study visuomotor adaptation and motor learning, showing that participants using a tablet reproduced similar learning curves and retention rates [19]. A second tablet-based (Android) software system designed to perform large-scale research outside of the laboratory illustrates various experimental protocols and tasks for quantifying and analyzing the potential mechanisms of motor behaviors and sensorimotor control [20]. The authors highlight the flexibility in their system to be fully customizable to the developer’s needs and research interests. Although these and other studies have investigated game-based clinical diagnostics and motor skill assessment methods [21-30], none thus far have been designed specifically for pediatric populations and data collection inside and outside of a traditional clinical or laboratory environment.

### Objective of This Study

For the current health problem of evaluating UL motor function, we developed a Gamified mHealth System (MoEvGame), a novel iPad-based tool consisting of games to assess and monitor UL motor function remotely without additional sensors or equipment. As an initial step in this research, we undertook a feasibility study with the following aims: (1) To design and develop the MoEvGame to capture UL movement, which consists of 5 games that examine theory-driven features of UL movement (whole-limb reaching, fine motor control, manual dexterity, and bilateral coordination) critical for performing functional tasks and daily activities. We introduced a software prototype for studying UL movement, consisting of an iOS app, a cloud-based data management and storage system, and a data analysis pipeline. Customizable features of the prototype allow for the design and implementation of game-based protocols considering different cohorts, age groups, and mechanisms of UL function. (2) To develop mathematical and analytical techniques to quantify spatiotemporal measures of whole-limb movement, fine motor precision, and bimanual coordination. As an additional novelty and highlight of MoEvGame, we included automated scripts (Multimedia Appendix 1) that are capable of performing real-time analysis of movement data collected with the iPad, thereby eliminating the need for time-consuming post-processing and analysis for movement quantification. (3) To collect preliminary data from typically developing children inside and outside a laboratory environment to show the prototype's initial feasibility and proof of concept.

This paper is structured as follows. In the Methods section, we describe the study participants, game prototype and description, data management, processing, and analysis methodologies. The Dependent Variables and Analytic Methods section briefly describes the analytical approach and algorithms to map spatiotemporal movement variables to motor performance. In the Statistical Analysis section, we present the statistical methods to compare speed-accuracy tradeoffs using the participant’s data. The Results section presents the motor performance data and correlation analysis. Then, the Discussion section describes the principal findings with limitations and opportunities of the MoEvGame. Finally, the paper concludes with planned future research directions and the translational potential of using mHealth in pediatric rehabilitation studies.

## Methods

### Participants

We collected data from 3 children in a laboratory setting and 28 children in a community setting (Discovery World Museum, Milwaukee, WI [31]) for initial feasibility testing of our prototype. The 3 children tested in the laboratory were run through a pilot and had to address any technical concerns before testing in a community setting. General guidance on how to complete each game was provided to each user. Anyone aged 5-14 years was eligible to participate. We used two Apple iPads (12.9” display, 7th generation) that were installed with MoEvGame. All participants were asked to play any of the games they preferred on the exhibited iPads. Research staff provided verbal instructions and cues if the participant did not understand or ignored the purpose of the game. Besides age, no other identifying information was collected from participants.

### Ethical Considerations

The experimental protocol of the study involving human participants was carefully designed and implemented in strict compliance with ethical standards. The Maquette University Institutional Review Board (IRB) approved the overall protocol for this research project (IRB number 3675). In this feasibility study, we collected only anonymous hand movement data from the iPad application and did not collect any personal identifying information from participants to ensure privacy and confidentiality. Therefore, the data were completely anonymous and a waiver of informed consent was granted by the IRB. No compensation was provided to participants.

### MoEvGame Prototype

We developed MoEvGame targeting the Apple iPad platform and a cloud-based data management system. The system harnesses features of mobile devices and gaming technology to study real-world movement in different environments without additional equipment or sensors. The tasks were designed to aim at four key features of skilled motor control: (1) whole limb reaching, (2) fine motor and individual finger control, (3) manual dexterity, and (4) bilateral coordination. These features are essential for performing activities of daily living and for play and leisure participation [32]. Furthermore, these domains map onto discrete but overlapping neural substrates underlying motor control. Whole-limb reaching involves a sensorimotor network (posterior parietal cortex, premotor, and primary motor cortices) to identify objects in space and move the end-effector to the object [33]. Individual finger movement requires the integrity of the fast-conducting corticomotor projections to control individual muscles in the hand [34]. Bilateral coordination requires cooperation between the motor systems facilitated by the corpus callosum [35].

### Game Descriptions

**Figure 1:**
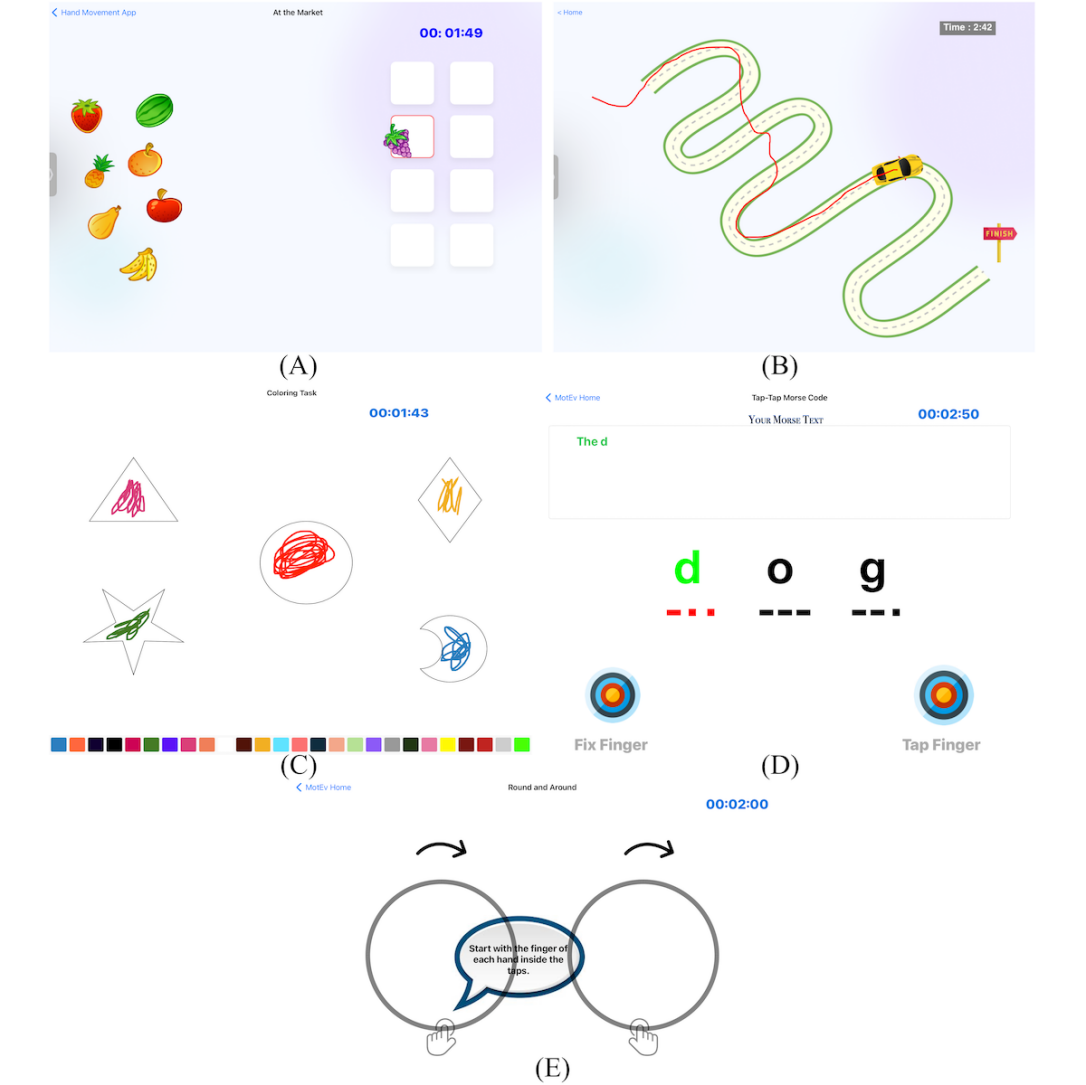
Representative user interface of movement games within MoEvGame. Together, the games test whole-limb movement (A and B), fine motor control and manual dexterity (C and D), and bilateral coordination (E).

MoEvGame was developed as illustrated in Figure 1, considering a kinematic approach to studying UL motor performance. While playing, spatiotemporal data were recorded continuously to evaluate multiple variables related to speed, accuracy, precision, coordination, and skill learning. Of note, a speed-accuracy tradeoff framework was incorporated for the game design. Each game requires accurate movements to be completed within a time limit. Thus, participants were required to complete the games within the time limit to get feedback and “credit” for completing the game.

### At the Market

At the Market involves using a finger to drag and drop fruits into crates. The border of each crate will turn green if placed accurately and red if an error occurs (part of the fruit remains outside the crate, left panel of Figure 1A). A trial of this game block consists of 2 attempts using the right and left hands, respectively, taking 60 seconds for each attempt.

### Race to the Finish

Race to the Finish involves using a finger to drag a race car along a curvilinear track (Figure 1B). An error occurs if the participant draws a direct path from the starting to the ending location to encourage adherence to the task. A trial consists of performing 3 races with each hand, where each race ends after 30 seconds.

### Filling In

This is a coloring task with the Apple Pencil to color 5 shapes in 2 minutes. Those shapes reflect various curvilinear and rectilinear spaces (Figure 1C). The coloring task challenges fine motor control and spatial awareness. Similar coloring tasks are part of standardized developmental assessments [36].

### Secret Message

The Secret Message involves tapping or holding a finger following a “dot” and “dash” signal of Morse Code to generate a hidden message while holding the contralateral finger in a fixed target. To test potential mirroring movements, the finger of the static hand must remain placed in a contralateral circular target (Figure 1D). A trial involves 180 seconds to perform tapping a secret message; after 90 seconds, the tapping finger and contralateral finger are required to switch.

### Round and Around

Round and Around involves drawing circles with both hands in 4 modes: in-phase congruent, antiphase congruent, in-phase mirrored, and antiphase mirrored. In-phase: starting finger positioning will be at the same location on the circle (eg, the top of the circle); antiphase: starting finger position will be 180 apart (eg, one finger at the top and a finger from the other hand at the bottom of the circle); congruent: movement direction is consistent (both fingers moving clockwise); and mirrored: movement direction is opposite (one finger moving clockwise and the other counterclockwise). Participants will trace around the circle for 30 seconds in each mode.

### Data Management, Processing, and Analysis

**Figure 2:**
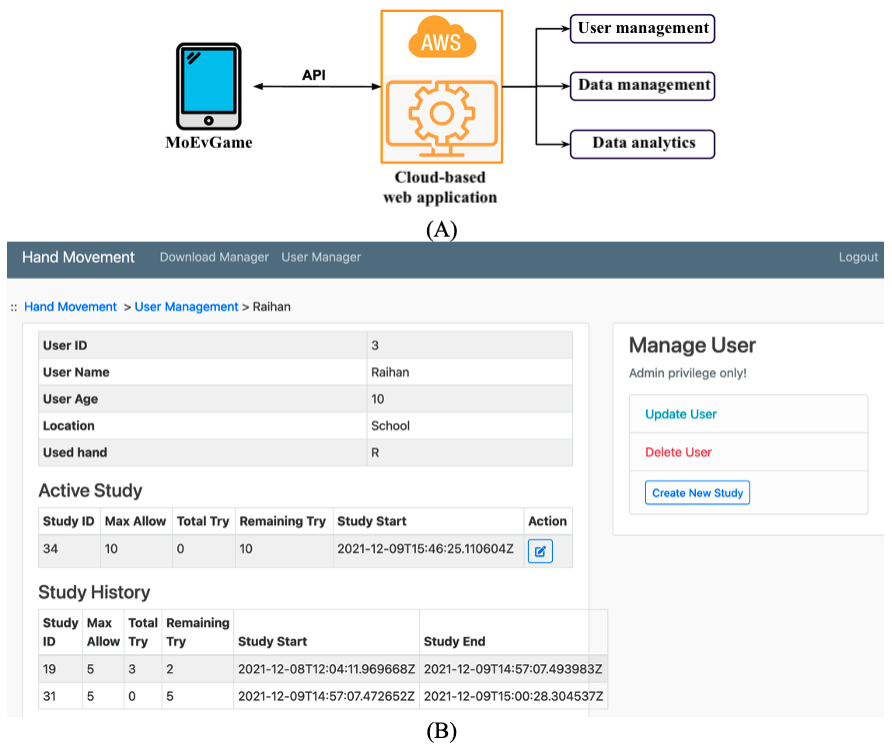
MoEvGame. (A) Prototype architecture. (B) Participant and game data management system in a web-based platform. API: application programming interface, AWS: Amazon Web Services.

A cloud-based data server has been designed and developed to manage the participants, game data storage, processing, and analysis (Figure 1). The architecture of the prototype is illustrated in Figure 2A, where the game data are transferred to a cloud server from the device via an application programming interface. The data and user management system prototype is implemented using the Python Django framework, JavaScript, and PostgreSQL database and deployed to an Amazon Web Service (AWS) EC2 cloud server. There are three core features of this management system as follows.

#### User Management

Each participant’s interaction with the app is controlled through a web-based dashboard. This is primarily used to link a participant to a study identified (eg, study ID) and to set how many attempts can be performed; this is necessary to limit practice and learning effects that might affect motor performance (Figure 2B). Moreover, the retention-based study design has been incorporated into the study management.

#### Data Management

The database has been designed by focusing on holistic data integration [37] that will enable data analysis from both individuals and specific groups. Data transmission depends on the data structure and storage representation. In the prototype, the raw data generated from games are mainly time series of spatiotemporal measures of the cursor position. Thus, we designed a database system with low latency to process a very high volume of JSON data [38]. Researchers can download user data from the dashboard once the tasks are completed for analysis and further study.

#### Data Analysis

While the raw data are similar, each game has specific performance measures and outcome data parameters requiring varied analytical techniques. Based on the objective of the game and its dependent variables, we devised specific statistical analysis plans and implemented these as Python scripts. Data were downloaded from the server in CSV format and analyzed accordingly to generate the results. The primary goal of the analysis was to demonstrate the feasibility of the prototype's automated and real-time analysis capabilities. The following section will describe the analysis plan for each game and validate it with representative participants' data.

### Dependent Variables and Analytic Methods

#### Whole-Limb Movement

**Figure 3:**
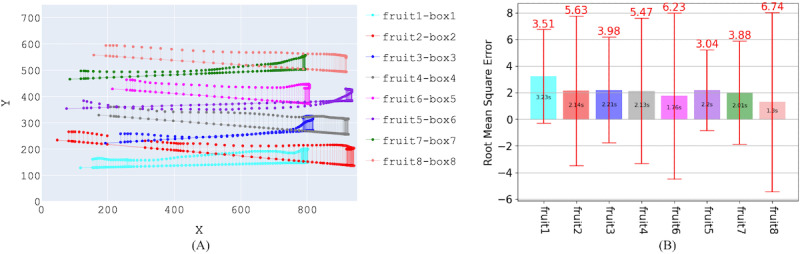
Analysis of At the Market game. (A) Movement trajectories for each item (a piece of fruit). (B) Root mean square error (red whisker) and completion time (bar) for each movement relative to the shortest path from item to target box.

Spatiotemporal variables such as movement path (x and y coordinates in 2D space) and time are used to quantify movement speed and accuracy. Dragging, dropping, and object tracking in the touchscreen were previously found to be effective methods to measure arm movement proficiency [39], where the completion time(s), accuracy, and deviation error from the shortest path trajectory (mm) were considered important dependent measures. In At the Market (Figure 3), suppose the movement path of an object (fruit) is (*x_i_*,*y_i_*); time is *t*_1_, where *i*=1, 2, ..., N, with N the total number of samples; *P*_1_ and *P*_2_ are the coordinates of the starting position of the fruit and the target destination (box), respectively. The perpendicular deviation of each point (*d_i_*) of the path from the *P*_1_*P*_2_ shortest path is calculated using equation (1). Then, the root mean square error (RMSE) of the whole movement path is calculated using equation (2).

Here, α, β, and δ are the shortest path (*P*_1_*P*_2_) coefficients. 



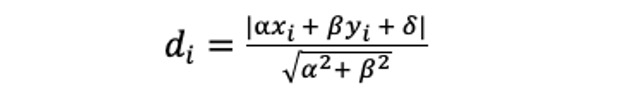



**(1)**



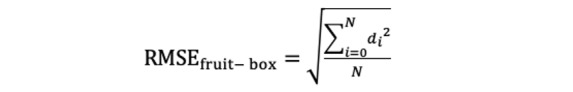



**(2)**

**Figure 4:**
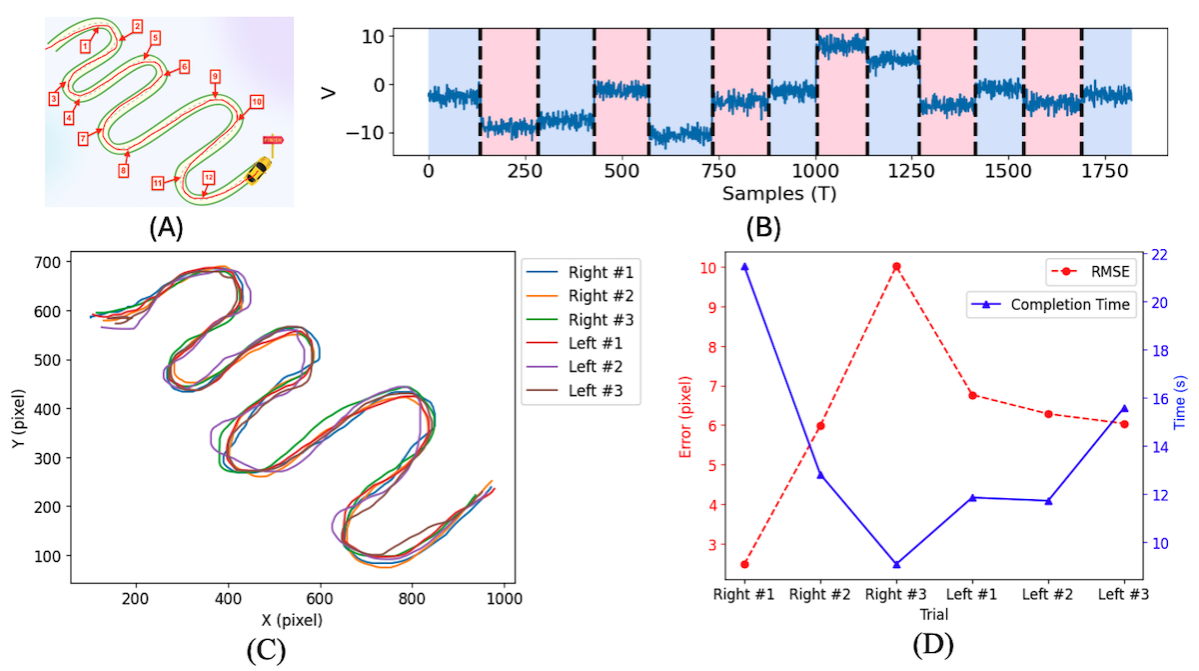
Analysis of Race to the Finish game. Changes in movement direction were determined using change point detection and the pruned exact linear time (PELT) method. (A) Break points in the curvature track were manually defined. (B) The PELT model detects the K=12 change points as noted by the change in criterion V. (C) Movement trajectories of 6 attempts. (D) Error (left y-axis) and completion time (right y-axis) are plotted for each attempt. Note the speed-accuracy trade-off for these 6 attempts.

The Race to the Finish task (Figure 4) tests visuomotor coordination of accurately tracing a curvilinear path. The first-order derivative (*dy*/*dx*) of the movement path has been used to identify the movement direction and segment to calculate the deviation error from the ideal path [40]. Let us consider a multivariate nonstationary first-order derivative signal *dy*/*dx = {dy*/*dx_1_, ..., dy*/*dx_T_}* that takes value in (– ≤ *dy*/*dx_i_ ≤* ) (and has T samples. The pruned exact linear time (PELT) method [41] identifies the ideal segmentation τ from a criterion *V* (τ, *dy*/*dx)* that is minimized for K, the known number of changes. In our case, equation (3) was minimized for K=12 (see Figure 4A) using the radial basis function (“rbf”) of the PELT method. Figure 4B shows the detection of τ=13 segments using K breakpoints from a movement path (x, y) of a participants’ trial.



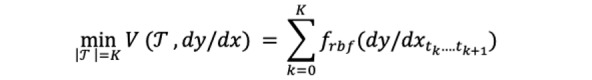



**(3)**

where *f*_rbf_(.) is a Gaussian kernel-based cost function that measures the goodness of fit of the nonparametric subsignal 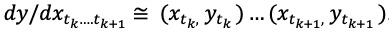 . The cost function *f*_rbf_ for a subsignal *z_a...b_* is shown in equation (4), where > 0 is the bandwidth parameter. The PELT minimization method identifies the segments of movement curvature from the criterion, *V*. These segments are used to calculate the error distance from the ideal racing track.



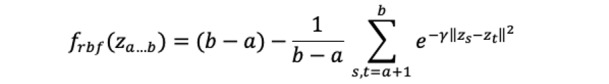



**(4)**

Each segment 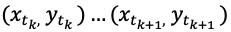. predicted by the PELT model has been used to calculate the perpendicular distance (*d_i_*) between the movement point (*x_i_*,*y_i_*) and ideal curvature using equation (5).



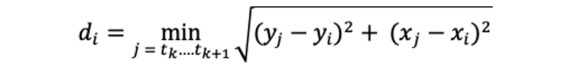



**(5)**

where *i*=1, 2, ..., M, and M is the number of samples in the ideal curvature of 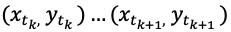. segment boundary. Figure 4C shows the movement path of 6 trials (3 with the right hand and 3 with the left hand) and Figure 4D shows the respective RMSE and completion time of each trial. A speed-accuracy trade-off is demonstrated by the negative correlation between completion time and error; greater accuracy requires greater control and, thus, longer completion time. Furthermore, we can observe that the participant of this trial (12 years) performed better in the Right #2 and Left #2 blocks (Figure 4D), whereas the Right #1 block took the longest to complete with the smallest RMSE.

#### Fine Motor Control and Manual Dexterity

**Figure 5:**
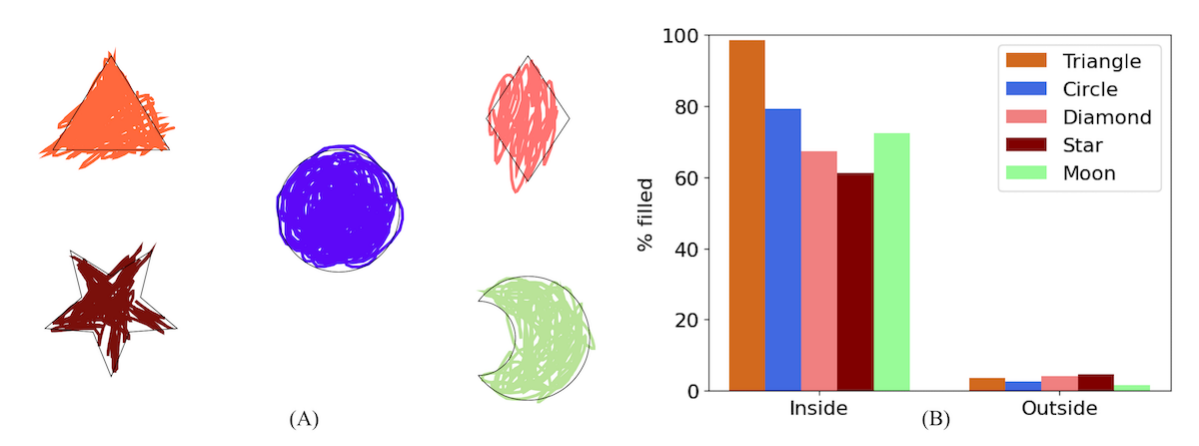
Analysis of Filling In game. (A) Sample coloring performance. (B) Bar plot of coloring accuracy, divided into the proportion colored inside and outside the target shape.

Individual finger and fine motor control are needed for object manipulation and handwriting. Drawing or coloring objects (eg, square, circle, or other shapes) and handwriting tasks are common techniques for evaluating fine motor precision and manual dexterity in children with developmental disabilities [42,43]. In the Filling In task (Figure 5), the accuracy (% filled inside the shape; % filled in outside the shape) and completion time are considered dependent measures of fine motor control. To compute these, we reconstructed the colored shape from the game data as illustrated in Figure 5A. The pixels colored inside or outside the target shape were calculated using their geometric properties (eg, triangle, circle, star). Since the participant’s movements during coloring likely contain duplicate pixel values, digital image processing techniques are applied to calculate the unique colored pixels from the reconstructed images. The percentage of coloring inside each shape is shown in Figure 5B, indicating the accuracy of fine motor control of an individual trial.

**Figure 6:**
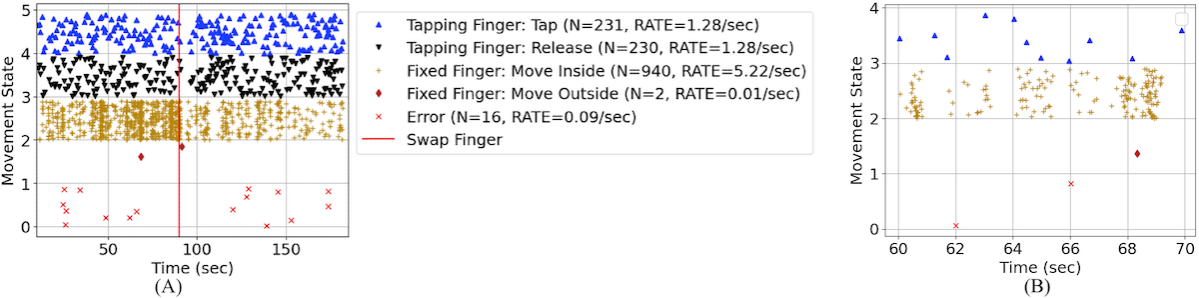
Analysis of Secret Message game. (A) Movement state of tapping finger and contralateral static (fixed) finger movement across time. (B) Magnified view of 10 seconds of tapping data. Note the user's non-tapping finger moved outside the target once (red diamond) and made two tapping errors (red x) with the tapping finger.

Speed and coordination of the distal UL correlate with corticospinal system integrity [44]. The Secret Message game (Figure 6) tests the motor performance of individual finger movements. Furthermore, because the contralateral (nontapping) finger is required to be engaged statically while the other finger is tapping, this game also tests response inhibition and mirroring movements. Tap and release speed, number of errors in tapping hand, and number of displacements (ie, nontapping finger movements inside or outside the target) are calculated as dependent measures. Errors are defined as inappropriate tap and release combinations corresponding to a “dot: or “dash” from Morse Code. For example, if a child pressed too long for a “dot,” this would be counted as an error. Figures 6A and 6B show the state of the tapping and nontapping fingers and the error rate with respect to time of a single trial in which the participant revealed 38 characters (right finger tapping 13 and left finger tapping 25 characters) of the secret message. In these figures, each data point represents a different state of the left and right fingers (eg, blue triangles represent the tapped state); data points within a state are offset in the figures for clarity. From these data, we observed that the tapping rate (blue triangle) increased, and the error rate (red x) decreased as the trial continued, perhaps indicating the participant understood the rules of the game. The amount of movement within the fixed finger target was less when the fingers were switched (gold plus symbols, states 2-3).

#### Bimanual Coordination

Bimanual coordination is important for completing many everyday activities. The Round and Around game is designed based on dynamical systems theory to challenge bimanual coordination with different levels of stability between the limbs [45,46]. We used 2 spatiotemporal measures to quantify bimanual coordination: (1) phase synchronization, which was defined as the instantaneous phase difference between the right hand and left hand during in-phase (ideal=0°) and antiphase (ideal=180°) conditions; and (2) speed synchronization, which was the difference in angular velocity of each hand. Let, *y*_1_=*x*(*t*_1_ )= sin(ω_1_ *t*_1_+ϕ_1_ ) and *y*_2_ =*x*(*t*_2_)= sin(ω_2_ *t*_2_+ϕ_2_) are 2 nonlinear signals of the right finger and left finger, respectively. Here, ω, *t*, and ϕ represent angular velocity, time, and initial phase, respectively. The instantaneous phase has been calculated from the argument of the analytical signal based on Hilbert Transform [47], as defined in equation (6).



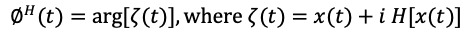



**(6)**

As *y*_1_ and *y*_2_ signals are sinusoidal, the Hilbert Transform method reconstructs the interpretable phases as 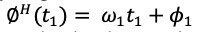 . and 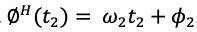., respectively. The phase difference between *y*_1_ and *y*_2_ has been estimated at a certain time, *t*, using equation (7), where *t* = *t*_1_ = *t*_2_.



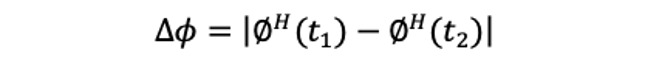



**(7)**

**Figure 7:**
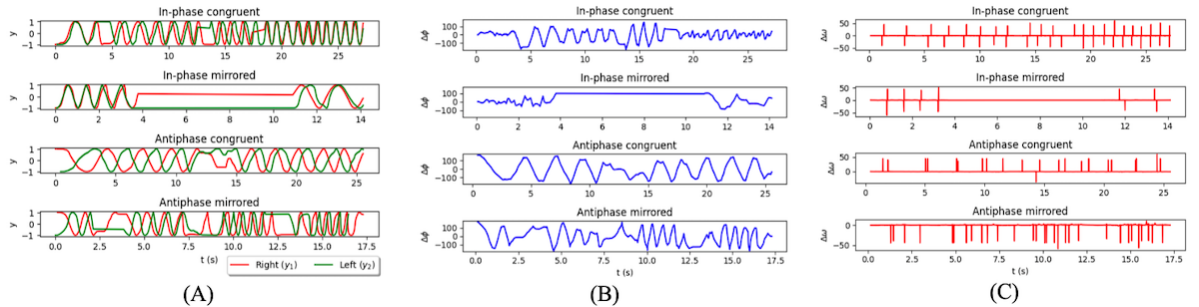
Analysis of Round and Around game. Each panel shows 4 plots corresponding to the 4 modes of coordination (in-phase congruent, in-phase mirrored, antiphase congruent, antiphase mirrored). (A) Raw angular position traces (*y*_1_ and *y*_2_) of each hand. (B) Phase difference (Δϕ) between *y*_1_ and *y*_2_. (C) Velocity difference (Δω) between *y*_1_ and *y*_2_.

The velocity difference (Δω) between *y*_1_ and *y*_2_ has been calculated at a certain time, *t*, using Δω= ω_1_-ω_2_, where *t* = *t*_1_ = *t*_2_. Figure 7 displays the bimanual hand position (A), phase difference (B), and velocity difference (C) from a representative participant.

### Statistical Analysis

As an initial proof-of-concept, we evaluated the feasibility of MoEvGame using 2 theory-based analytic approaches. First, we used a speed-accuracy tradeoff framework to compare motor performance [48]. In nonlearned tasks, users balance basic parameters of speed and accuracy to complete a task [49]. In the procedure, participants were not given specific instructions to use a “speed first” or “accuracy first” strategy. Therefore, different strategies that optimized movement speed and accuracy were likely employed. From this speed-accuracy framework, we predicted that participants moving faster would also show higher errors or error rates. In contrast, if both speed and accuracy were optimized, we concluded the participant performed a highly learned task. The speed-accuracy framework was used to analyze the data from At the Market, Race to the Finish, and Secret Message.

At the group level, analysis was performed based on developmental changes in motor abilities that would be expected as children mature. Thus, for a given assessment of motor development, one would expect to find age-related improvements in motor performance. A group analysis using the Pearson correlation coefficient was performed by combining data for each age and examining the relationship between changes in age and motor performance.

For bimanual coordination, a separate analysis was used based on the theoretical differences in difficulty for performing in-phase versus antiphase movements. Here, we examined the mean, standard deviation, and 95% confidence interval of the phase and angular velocity difference between the hands as measures of performance for each trial. We termed this “phase synchronization” or “speed synchronization,” respectively, where values closer to 0 represent coherence between the two hands.

## Results

### Participants’ Characteristics and Feasibility

A total of 56 trials were conducted by 31 participants. The participants’ median age was 9.0 (IQR 5.0-14.0) years, where most were third- or fourth-grade students (n=18, 58.06%, 9≤age≤11). Some participants attempted more than one of the 5 games, whereas some just tried a single task. The summary of the trials and participation is detailed in Table 1. General observations during testing showed that children were willing and excited to try the games and were comfortable interacting with the iPad. A small percentage of children did not complete the entire game, and we excluded the incomplete trials from the analysis.

**Table 1:** Participant characteristics and engagement.

			Participants (N=31)
**Characteristics**			
	Age (years), median (IQR)	9.0 (5.0-14.0)	
	Second or lower grade student, n (%)	9 (29.04)	
	Third- or fourth-grade student, n (%)	18 (58.06)	
	Fifth- or higher grade student, n (%)	4 (12.9)	
**Trials, n (%)**			
	At the Market	11 (19.6)	
	Race to the Finish	21 (37.6)	
	Filling In	2 (3.5)	
	Secret Message	13 (23.2)	
	Round and Around	9 (16.1)	

### Motor Performance and Correlation Analysis

**Figure 8:**
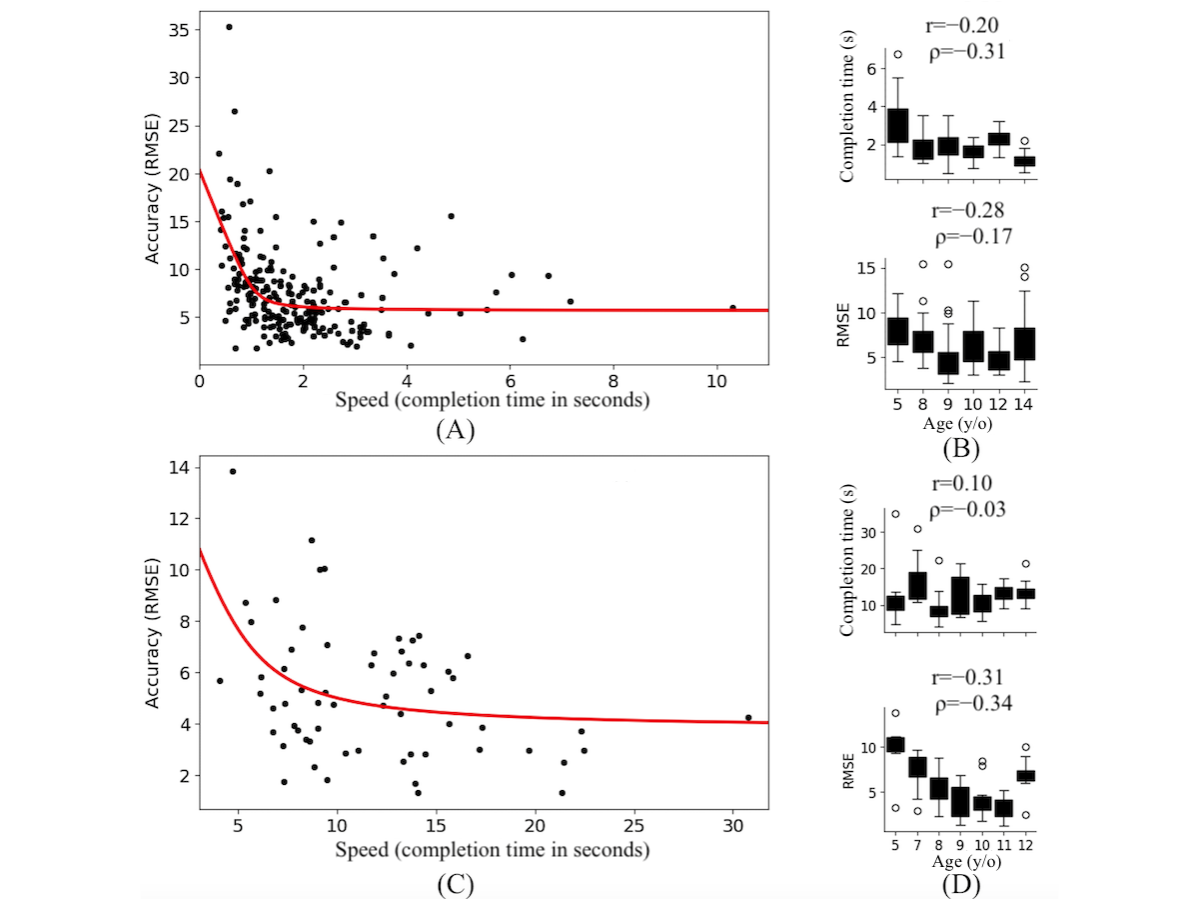
Whole-limb movement assessment of group data. Plots (A, C) show movement speed versus accuracy, while plots (B, D) show the completion time and error (RMSE) as a function of the user’s age when playing At the Market (A-B) and Race to the Finish (C-D). Note the general speed-accuracy trade-off across the group and the negative correlation of spatial effort with age. The red curve is an exponential best fit to the points. RMSE: root mean square error.

Figure 8 shows the whole-limb movement performance on two games, At the Market and Race to the Finish, and their association with age. These games had the most participants (N=23, 74.2%) across the widest age range. Among these data sets, 21.7% of participants played both games, whereas 60.8% and 17.5% of trials were Race to The Finish and At the Market trials, respectively. We found that there was a negative correlation between speed and accuracy in At the Market (*r*=–0.42, ρ=–0.29) and Race to the Finish (*r*=–0.30, ρ=–0.35) as illustrated in Figure 8A and 8C, respectively. The area under the curve of the quadric curve in these figures indicated that low RMSE (high accuracy) could be achieved with optimal speed. Participant’s movement error (RMSE) decreased with increasing age (Figure 8B and 8D), indicating older participants had better movement accuracy.

**Figure 9:**
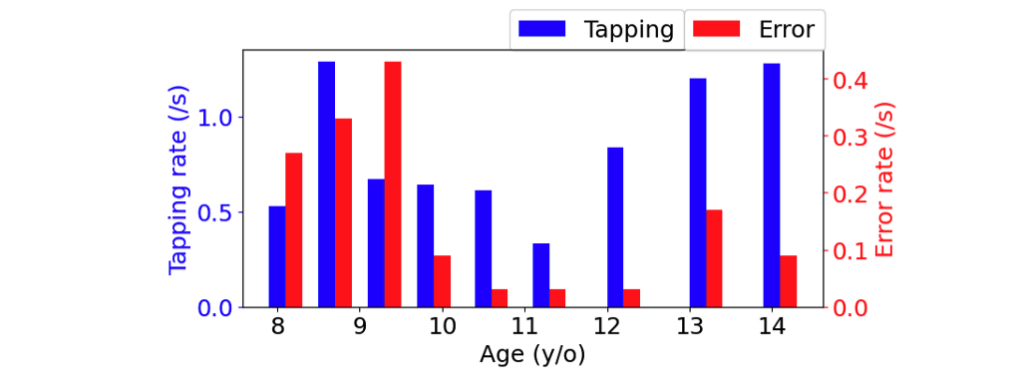
Tapping and error rate compared to user age for Secret Message. The finger tapping rate (speed) increased and the error rate (accuracy) decreased with increases in age.

Fine motor control and manual dexterity (finger movement) were evaluated from the speed-accuracy trade-off of Morse code finger tapping and error rates. The tapping rate was analyzed with respect to participants’ age, as shown in Figure 9. While both older and younger participants exhibited higher tapping rates, error rates were higher in younger compared to older participants.

Finally, bilateral coordination results from the group data are shown in Table 2. The mean (μ) and standard deviation of phase synchronization for in-phase coordination were less than from antiphase coordination. Likewise, left- and right-hand speed was higher for in-phase than antiphase coordination. Moreover, the right hand traveled a shorter total distance during antiphase congruent (mean 3.31, SD 1.67 m; coefficient of variation [CV]=0.51) and mirrored (mean 3.21, SD 1.67 m; CV=0.52) compared to in-phase congruent (mean 5.26, SD 3.04 m; CV=0.58) and mirrored (mean 4.04, SD 3.39 m; CV=0.84) coordination. These results indicate participants performed better and completed more full revolutions per second in the in-phase coordination, consistent with prior observations that antiphase bilateral movements are more difficult to coordinate than in-phase [50,51].

**Table 2:** Bimanual coordination outcomes from Round and Around game.

Mode	Phase synchronization (degree)			Speed synchronization (revolutions per second)							
	SD	Mean (95% CI)	Right hand				Left hand			Difference	
			SD		Mean (95% CI)	SD		Mean (95% CI)	SD		Mean (95% CI)
In-phase congruent	18.97	28.85 (28.49 to 29.21)	5.48		7.11 (7.02 to 7.21)	5.52		7.64 (7.54 to 7.74)	5.44		–0.52 (–0.62 to –0.43)
In-phase mirrored	16.07	23.78 (23.46 to 24.10)	4.97		7.60 (7.50 to 7.69)	4.97		7.24 (7.14 to 7.33)	5.02		0.36 (0.26 to 0.45)
Antiphase congruent	25.82	112.64 (111.98 to 113.29)	3.42		4.70 (4.63 to 4.77)	3.65		4.55 (4.48 to 4.63)	3.73		0.14 (0.06 to 0.22)
Antiphase mirrored	28.20	121.39 (120.53 to 122.24)	4.97		6.27 (6.15 to 6.39)	5.36		6.63 (6.50 to 6.77)	5.27		–0.36 (–0.49 to –0.23)

## Discussion

### Principal Results

This study aimed to demonstrate the feasibility of an mHealth app to quantify and evaluate UL movement. Pilot testing showed that MoEvGame can capture UL movement performance that is linked to aspects of motor control and development in children. Specifically, speed-accuracy tradeoffs were noted for At the Market, Race to the Finish, and Secret Message (Figures 8 and 9). These effects were observed because we did not ask participants to prioritize speed over accuracy, and thus, various strategies may have been used to complete the games. Notably, the speed-accuracy tradeoff is evidence for face and content validity, showing that the games test motor control processes and are suitable tasks for future studies of UL motor functions. In addition, age-related changes in UL performance were evident: in general, younger participants demonstrated higher spatial errors than older participants, consistent with the maturation and development of the motor system. These findings agree with prior results [52,53], where speed and accuracy were key factors in fine motor performance to complete a gamified toy. Other motor constructs, such as bimanual coordination, were examined. We observed differences in the left-hand and right-hand phases and speed synchronization between the four modes (in-phase or antiphase, congruent or mirrored) for the bimanual game that replicated prior studies in which in-phase coordination is more stable and therefore can be performed more efficiently than antiphase coordination [54]. These results are a promising first step toward validating this technology as an objective and standardized UL assessment tool.

Portable technology, such as mobile devices, advances modern technological research testing and protocol design. In a study by Mollà-Casanova et al [55], the authors implemented a series of tasks on an iPad for adults with stroke to test hand motor function and demonstrated strong correlations between variables measured with the iPad and outcomes from standardized clinical assessments. A host of other examples using tablets and mobile technology to study UL movement in children also exist in the literature [56]. However, some common limitations of these studies were object manipulation on a touchscreen, offline data analysis, real-world usability, and age-appropriate game design. The hand movement application described by Matic and Gomez-Marin and the portable motor learning laboratory described by Takiyama and Shinya [19] provide a strong foundation for using tablet devices to study motor control. Although these examples were not specifically designed for children, they could be adapted for pediatric populations. MoEvGame expands upon these ideas by adding gamification, automated analysis pipelines, and a data server connected to the application programming interface to facilitate data transfer and remote access.

Collectively, MoEvGame and other mHealth apps allow research to be conducted in contexts and environments. A common concern is that a participant’s function observed in traditional laboratories or clinic settings may be different in their home or other environments. With mobile assessment technology, this means assessments might occur at home or when studying pediatric populations at school. Studies that collect multiple data points spread out across times and environments would demonstrate the benefits of mHealth technology and showcase robustness not commonly available with laboratory-based assessments. Overall, investigations that employ continuous monitoring of UL movements in varied environments will lead to more impactful and ecologically valid translational research.

### Limitations and Opportunities

There are limitations and future variables to consider before further validating MoEvGame. First, the feasibility testing data were collected from a convenience sample of children attending a public museum exhibit. Testing in a larger and more representative sample of children would allow us to enhance the generalizability of our findings and begin to explore age-normative values. Second, since we were interested in learning how children interacted with MoEvGame, no standardized instructions were provided, resulting in varied strategies to achieve the goal of each game. For instance, while playing At the Market, some users moved the fruit directly to the box, while others moved it underneath and placed it into the crate from the opposite side. Considering we do not have other measures of motor development or function for comparison, overall this limits the interpretation of the MoEvGame data and how data from an individual child relates to their motor function. Considering these user behaviors is critical for further development and refinement of the MoEvGame. Finally, by using a 2D touchpad, we are limited to measuring the final endpoint of movement rather than quantifying UL movement kinematics in 3D, similar to what is obtained from laboratory motion capture systems. We plan to compare the MoEvGame movement variables to 3D kinematic variables in future validation studies. In spite of these limitations, we were able to achieve our primary objective of demonstrating feasibility and proof-of-concept. The goal of future work will be to systematically test the validity and reliability of MoEvGame in children with and without motor impairments with a large sample and wider age group. However, age-related changes can affect motor behavior [57], specifically older kids or adolescents with motor impairment who are capable of using a touchscreen device. Therefore, it may be necessary to capture an inclusive data set for further investigation.

### Conclusions

In this feasibility study, we describe a gamified mHealth system, MoEvGame, as a tool to study UL movement in children with and without neuromotor impairments. Valid and objective outcomes are pivotal for clinical trials and provide clinicians with relevant information about a child’s motor function and ability. Moving beyond traditional assessments that are time-consuming and require in-person administration, our approach for assessing UL movement skills incorporates gamification into ubiquitous and accessible technology. Research on the mechanisms of UL motor dysfunction is critical for advancing evidence-based assessment and treatment of children with motor impairments. The availability of fast, flexible, and objective testing tools using common mobile devices will be advantageous to researchers, clinicians, and families. Future work in participants with and without motor impairments across a wide age range in both laboratory and nonlaboratory settings will test the reliability and validity of MoEvGame.

## Appendix

Multimedia Appendix 1 Dataset and codebase.

## Abbreviations

CV: coefficient of variation
IRB: Institutional Review Board
MoEvGame: Gamified mHealth System for Upper Limb Motor Evaluation
mHealth: mobile health
PELT: pruned exact linear time
rbf: radial basis function
RMSE: root mean square error
UL: upper limb

## Data Availability

The anonymous datasets generated and analyzed during this study are available in the GitHub repository [58].
