# Supplementary figures and images for: Gamified mHealth System for Evaluating Upper Limb Motor Performance in Children: Cross-Sectional Feasibility Study
Source: JMIR Serious Games. 2025 Feb 28;13:e57802. doi: 10.2196/57802 (PMC11909489; doi:10.2196/57802)

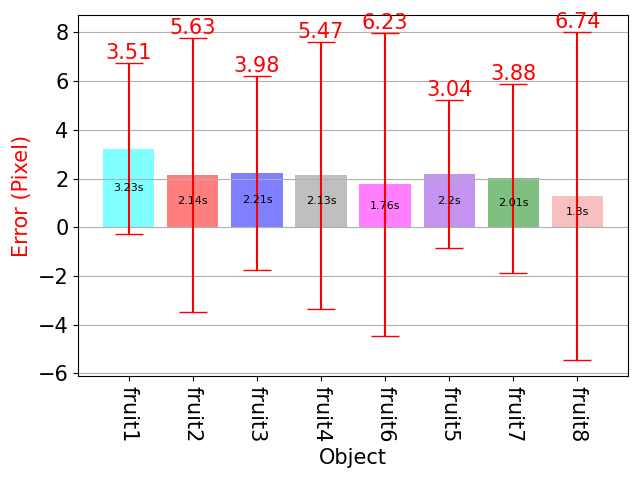

Supplement: Multimedia Appendix 1 [file games_v13i1e57802_app1.zip › FeasibilityTesting/At The Market/Root-mean-square deviation of a try.png]

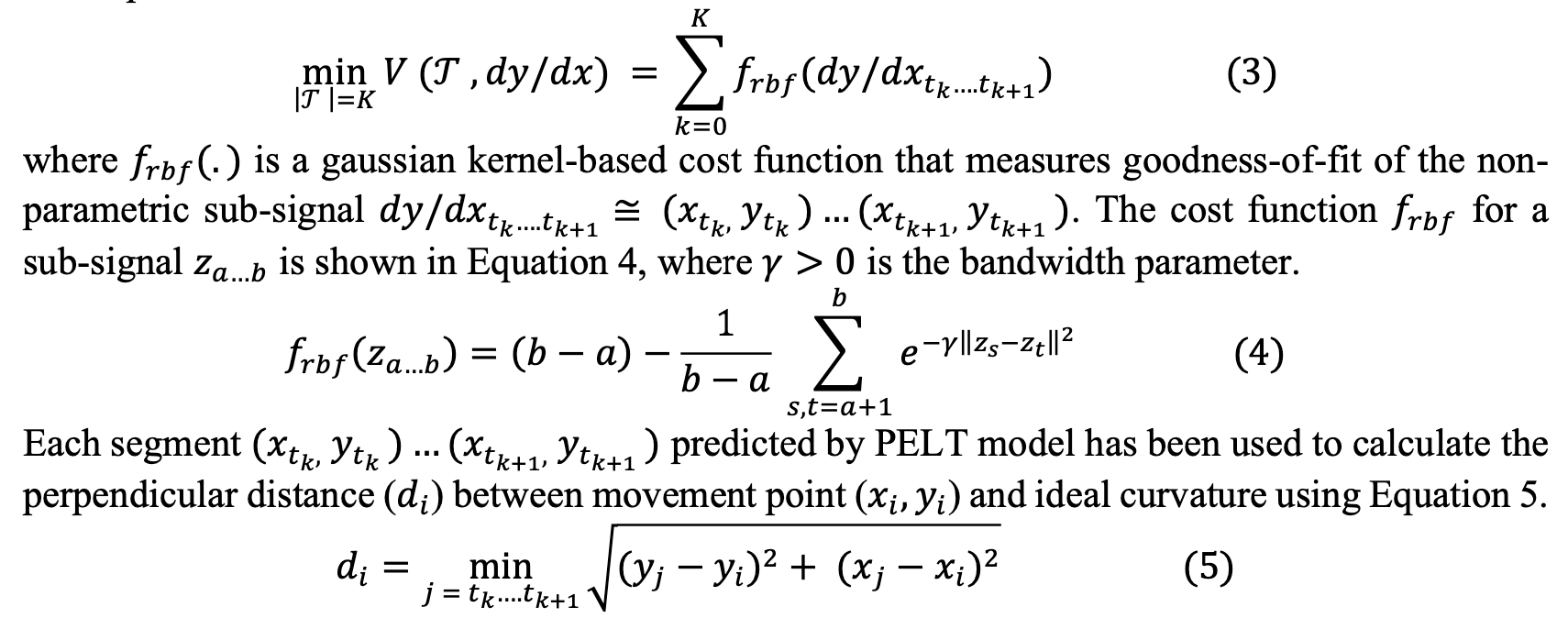

Supplement: Multimedia Appendix 1 [file games_v13i1e57802_app1.zip › FeasibilityTesting/Racing/PELT_Method.png]
